# Sporadic ALS is not associated with *VAPB *gene mutations in Southern Italy

**DOI:** 10.1186/1477-5751-5-7

**Published:** 2006-05-29

**Authors:** Francesca Luisa Conforti, Teresa Sprovieri, Rosalucia Mazzei, Carmine Ungaro, Alessandro Tessitore, Gioacchino Tedeschi, Alessandra Patitucci, Angela Magariello, AnnaLia Gabriele, Vincenzo Labella, Isabella Laura Simone, Giovanni Majorana, Maria Rosaria Monsurrò, Paola Valentino, Maria Muglia, Aldo Quattrone

**Affiliations:** 1 Institute of Neurological Sciences, National Research Council, Mangone, Cosenza, Italy; 2 Second Division of Neurology, Second University of Naples, Naples, Italy; 3 Department of Neurology and Psychiatry, University of Palermo, Palermo, Italy; 4 Department of Neurological and Psychiatric Sciences, University of Bari, Bari, Italy; 5 Department of Neurosciences, Psychiatric and Anaesthesiological Sciences, University of Messina, Messina, Italy; 6 Institute of Neurology, University Magna Graecia, Catanzaro, Italy

## Abstract

Mutations in the Cu/Zn superoxide dismutase (*Sod1*) gene have been reported to cause adult-onset autosomal dominant Amyotrophic Lateral Sclerosis (FALS). In sporadic cases (SALS) de novo mutations in the *Sod1 *gene have occasionally been observed. The recent finding of a mutation in the VAMP/synaptobrevin-associated membrane protein B (VAPB) gene as the cause of amyotrophic lateral sclerosis (ALS8), prompted us to investigate the entire coding region of this gene in SALS patients. One hundred twenty-five unrelated patients with adult-onset ALS and 150 healthy sex-age-matched subjects with the same genetic background were analyzed.

Genetic analysis for all exons of the *VAPB *gene by DHPLC revealed 5 variant profiles in 83 out of 125 SALS patients. Direct sequencing of these PCR products revealed 3 nucleotide substitutions. Two of these were found within intron 3 of the gene, harbouring 4 variant DHPLC profiles. The third nucleotide variation (Asp130Glu) was the only substitution present in the coding region of the *VAPB *gene, and it occurred within exon 4. It was found in three patients out of 125. The frequency of the detected exon variation in the *VAPB *gene was not significantly different between patients and controls. In conclusion, our study suggests that *VAPB *mutations are not a common cause of adult-onset SALS.

## Findings

Amyotrophic Lateral Sclerosis (ALS) is a progressive lethal disorder of motor neurons of the spinal cord and brain. More than 90% of ALS patients are sporadic (SALS), not showing any familial trait. Approximately 10% of cases are familial (FALS), and within these lie several distinct forms of the disease. The most common form of FALS is autosomal dominant for which to date 2 genes have been identified: ALS1, with adult-onset caused by mutations in the gene encoding the cytosolic antioxidant enzyme Cu, Zn-superoxide dismutase (*Sod1*) [1,2]; and ALS4, a rare juvenile-onset disorder, associated with mutations of the senataxin gene (*SETX*) [3]. An autosomal recessive, juvenile-onset form (ALS2), has been associated with mutations of the *Alsin *gene located at 2q33 [4]. To date over 100 different missense mutations in the *Sod1 *gene [5] and up to eight described mutations in the *Alsin *gene have been reported [6].

By contrast, sporadic ALS is believed to be a multi-factorial disease in which modifying genes and environmental agents affect its clinical manifestation, but few associated genes have so far been identified [7-13]. De novo mutations in the *Sod1 *gene have only occasionally been observed in sporadic cases of ALS suggesting that mutations in this gene are a rare cause of non familial forms of ALS [14,15].

The finding by Nishimura et al.[16] of a missense mutation in the VAMP/synaptobrevin-associated membrane protein B (*VAPB*) gene in autosomal dominant motoneuron diseases (ALS8), prompted us to investigate the entire coding region of this gene in 125 SALS patients.

Following informed consent, blood was taken from 125 unrelated Caucasian patients from Southern Italy, who fulfilled the El Escorial criteria [17] for ALS. Samples were also taken from 150 controls, matched for age, sex and geographic region. Clinical information is provided in Table 1. Lymphocyte DNA was extracted from blood samples using standard procedure. *Sod1 *mutation-negative patients were screened using primer flanking the intron-exon regions of the *VAPB *gene. The PCR products were analyzed by Denaturing High Performance Liquid Chromatography (DHPLC) on a WAVE Nucleic Acid Fragment Analysis System (Transgenomic Inc., Mountain View, CA). Representative samples with abnormal profile in DHPLC were sequenced by the ABI Prism BigDye Terminator cycle sequencing ready reaction kit (Perkin Elmer- Applied Biosystems Inc., Foster City, CA).

DHPLC analysis of the *VAPB *gene [GenBank accession no. AL035455] detected five variant profiles in 83 out of 125 SALS patients. Direct sequencing of PCR products revealed 3 nucleotide substitutions. Two nucleotide substitutions, g.134585C>T and g.134688A>G were found within intron 3 of the gene, harbouring 4 variant DHPLC profiles, because of their combination in homozygous or heterozygous states. These polymorphisms were already present in the SNP database as rs 2234487 and rs 2234488, respectively [18].  The frequencies in our SALS patients and in control population were 65% vs 64% for the rs 2234487 and 42% vs 45% for the rs 2234488, respectively.

The third nucleotide substitution was the only substitution present in the coding region of the *VAPB *gene and it occurred within exon 4 at nucleotide g.138864T>G (Asp130Glu).

The Asp130Glu variation is located between MSP and Coiled-coil domains, and involves an aminoacid residue of aspartic acid not conserved among different species. We identified this variation in heterozygous state in three out of 125 SALS patients (2.0%) and in three of 150 (2.4%) sex-age-matched healthy subjects from the same geographical area, suggesting that this aminoacidic substitution is not causative of the disease.

The data presented herein suggest that *VAPB *mutations are not a common cause of adult-onset SALS in Italian population. Further, the novel nucleotide variation identified in exon 4 of *VAPB *gene represents a polymorphism, probably linked to a restrict geographic area.

## Authors' contributions

FLC conceived of the molecular study and participated its design and coordination and wrote the manuscript. RM and MM participated in the design of study and helped to draft the manuscript. AT, GT, MRM, VL, ILS, GM, PV, provided the samples and performed clinical diagnosis of the patient group from Naples, Palermo, Bari, Messina and Catanzaro, respectively. TS and CU carried out the molecular genetic study and performed DHPLC analysis. AP, AM, and ALG participated in carrying out the sequencing. AQ conceived of the study and participated its design and coordination and helped to draft the manuscript. All authors read and approved the final manuscript.

**Table 1:** Clinical information of the studied populations

**Subject**	**Total**	**Male/Female**	**Age at Onset^a ^(years)**	**Mean Age**	**Spinal Onset**	**Bulbar Onset**
SALS^b^	125	66/59	54.9 (40–80)	58.9 (41–88, SD 10.5)	106	19
Controls	150	69/81	-	59.9 (40–78, SD 12.2)	-	-

^a ^Mean age of onset (with lowest/highest and Standard Deviation values in parentheses);

^b ^SALS: Sporadic Amyotrophic Lateral Sclerosis
